# Eleutheroside B alleviates oxidative stress and neuroinflammation by inhibiting the JAK2/STAT3 signaling pathway in a rat high altitude cerebral edema model

**DOI:** 10.3389/fphar.2024.1506483

**Published:** 2024-11-15

**Authors:** Yacong He, Hongying Zhang, Xiu Zhang, Yue Han, Huxinyue Duan, Wenqian Song, Qingqing Tian, Yilan Wang, Guang Li, Chunjie Wu, Zhenxing Wang, Tianzhu Zhao

**Affiliations:** ^1^ State Key Laboratory of Southwestern Chinese Medicine Resources, School of Pharmacy, Chengdu University of Traditional Chinese Medicine School of Pharmacy, Chengdu University of Traditional Chinese Medicine, Chengdu, China; ^2^ Pneumology Department, Qujing Hospital of Traditional Chinese Medicine, Qujing, China; ^3^ Teaching and Research Office of Traditional Chinese Medicine Internal Medicine, Hospital of Chengdu University of Traditional Chinese Medicine, Chengdu, China; ^4^ Respiratory and Critical Care Medicine Center, Traditional Chinese Medicine Hospital of Meishan, Meishan, China

**Keywords:** high altitude cerebral edema, eleutheroside B, oxidative stress, neuroinflammation, JAK2/STAT3 signaling pathway

## Abstract

**Background:**

High altitude cerebral edema (HACE) is a condition where the central nervous system experiences severe impairment as a result of sudden oxygen deprivation at high elevations. At present, effective measures for preventing and treating this condition are still lacking. Eleutheroside B (EB), the primary natural active compound found in the *Eleutheroside senticosus*, has demonstrated various biological functions. It has also shown significant potential in addressing acute mountain sickness and various neurological disorders. However, additional investigation is required to explore the potential protective effects and its underlying mechanisms of EB on HACE.

**Methods:**

The male rats received pre-treatment with either vehicle, EB 100 mg/kg or 50 mg/kg, Dexamethasone 4 mg/kg, or coumermycin A1 100 μg/kg. To simulate the hypobaric hypoxia environment at a plateau of 6,000 m, a hypobaric hypoxia chamber was utilized. The therapeutic effects of EB were assessed through measurements of brain water content, histopathological observation, and evaluation of oxidative stress and inflammatory factors using immunofluorescence and ELISA. Furthermore, molecular docking, molecular dynamics simulation and Western blot were employed to clarify its molecular mechanism. Through these analyses, the underlying mechanism by which EB on HACE was identified.

**Results:**

Pre-treatment with EB demonstrated a significant protective effect against HACE by effectively reducing brain water content, down-regulating HIF-1α and AQP4 protein expression induced by hypoxia and reversing pathological changes in brain tissue and neuron damage. Compared to the group treated with HACE alone, the group pre-treated with EB showed a significant reduction in levels of ROS and MDA, as well as an increase in GSH. In addition, pre-treatment with EB led to a significant decrease in the levels of IL-1β, IL-6, and TNF-α. Molecular docking and dynamics simulations indicated that EB has a strong binding affinity to the JAK2/STAT3 signaling pathway. Western blot further confirmed that EB significantly downregulated the expression of JAK2/STAT3 related proteins in the brain tissue of HACE rats. Additionally, coumermycin A1, an agonist of the JAK2, reversed the anti-oxidative stress and neuroinflammation against HACE of EB.

**Conclusion:**

EB exerts its antioxidant stress and anti-neuroinflammatory effects by inhibiting the JAK2/STAT3 signaling pathway in a rat HACE model.

## 1 Introduction

Due to the rapid advancement of modernization and the continuous expansion of human activities in recent years, high-altitude plateau regions have become increasingly popular among tourists, workers, and scientific researchers. The environment is defined by a unique mix of decreased atmospheric pressure, limited oxygen levels, frigid temperatures, and strong UV radiation which significant challenges to human health (Moore et al., 1986). In particular, high altitude cerebral edema (HACE) presents significant hidden risks to public health due to its sudden onset, high mortality rate, and complex pathophysiological features (Johnson and Luks, 2016). However, in-depth understanding of the pathogenesis and effective measures for preventing and treating this condition are still lacking. Currently, there is a general consensus that the onset of HACE is mainly caused by changes in cellular metabolic functions and heightened vascular permeability, both of which result from cerebral hypoxia at higher elevations (Bailey et al., 2004; Purushothaman et al., 2008). These response mechanisms are primarily modulated by oxidative stress, which is crucial in initiating a series of chain reactions. Under low oxygen levels at elevated altitudes, there are disruptions in cellular energy processing, a transition towards heightened anaerobic metabolism, intensified oxidative pressure, and the generation of different oxidative byproducts such as reactive oxygen species (ROS) (Bailey et al., 2006). These reactive species can damage brain cell membranes and organelles, particularly mitochondria, causing cellular edema and dysfunction, which culminates in HACE. Simultaneously, the increased oxidative stress could also induced by the escalation of various pro-inflammatory agents, worsening the cerebral edema (Hackett and Roach, 2004). Consequently, it is of critical importance to explore pharmacological interventions or strategies that effectively mitigate oxidative stress to prevent and manage high-altitude cerebral edema.

There is an inflammation of the nervous system, in general, and of the nervous tissue in particular, called neuroinflammation. Recent studies have demonstrated that HACE is strongly associated with neuroinflammation, but the signaling pathway of neuroinflammation induced by altitude hypoxia remains unclear. The JAK2/STAT3 pathway is significantly involved in controlling oxidative stress and inflammatory response, especially in the cerebral cortex and hippocampus of central nervous system (CNS) (De-Fraja et al., 1998). Key components of this signaling pathway include Janus tyrosine kinases (JAKs) and signal transduction agonists of transcription proteins (STATs). Upon ligand binding to gp130 receptor subunits, a series of classical signaling cascades are initiated, leading to autophosphorylation of JAKs and subsequent phosphorylation of STATs (Liao et al., 2013). Notably, the induction of JAK2/STAT3 and oxidative stress are significantly correlated and mutually influence each other. When inflammatory cells are stimulated, the increase in inflammatory factors can activate the JAK2/STAT3 signaling pathway (Boengler et al., 2008), induces production of enzymes involved in ROS, results in elevated local ROS levels and leads to occurrence of oxidative stress (De-Fraja et al., 1998). Alternatively, excessive ROS accumulation can also lead to the activation of the JAK2/STAT3 signaling pathway (Cao et al., 2020). In neurological disorders, over-activation of the JAK2/STAT3 signaling pathway and accumulation of ROS lead to oxidative stress damage in neurons, mitochondrial dysfunction, and cell apoptosis, all contributing to neuronal damage and loss of neurological function (Panda et al., 2024). Given the crucial role of the JAK2/STAT3 pathway in regulating oxidative stress and inflammatory responses, it is expected that targeting this pathway may provide new insights and strategies for preventing and treating HACE and related diseases.

In the pursuit of effective treatment strategies for high altitude diseases, particularly HACE, traditional Chinese medicine has gained increasing recognition within the scientific community (Huang et al., 2024; Liu et al., 2024). *Eleutherococcus senticosus*, also named as Ciwujia, is a dual-purpose herbal medicine in the Aracanaceae family that can be used for both food and medicine. It has been traditionally utilized in TCM for its abilities to combat fatigue and improve memory (Gerontakos et al., 2021). Modern pharmacological studies further validates the significant neuroprotective (Xie et al., 2015), anti-inflammatory (Li et al., 2016), and anti-oxidant (Orellana-Urzúa et al., 2020) properties of *Eleutherococcus senticosus*. Eleutheroside B (EB), a key component of *Eleutherococcus senticosus*, exhibits noteworthy pharmacological effects including anti-oxidation (Kim et al., 2010), anti-fatigue (Cheng et al., 2023) and neuroprotection (Yang et al., 2010). Our previous study have shown that EB against HACE were associated with the inhibition of ferroptosis and necroptosis through Nrf2-antioxidant response signaling, inhibiting the progression of acute mountain sickness (Wang et al., 2022). However, the pharmacological role and underlying mechanisms of EB in HACE are unclear.

Based on the aforementioned background, our study proposes that EB may exert effects on anti-oxidant stress in the prevention and treatment of HACE by modulating the JAK2/STAT3 signaling pathway. To test this hypothesis, a rat model of HACE was established and the therapeutic effects of EB were evaluated through measurements of brain water content, histopathological observation, assessment of oxidative stress and inflammation factors. Additionally, molecular docking, molecular dynamics simulation, and Western blotting were employed to elucidate its molecular mechanism, thereby providing novel scientific evidence and potential therapeutic targets for HACE prevention and treatment. This study not only holds significant scientific value, but contributes positively to the advancement of plateau medicine.

## 2 Materials and methods

### 2.1 Reagents and antibodies

Eleutheroside B (purity ≥99.96%, MUST-24061302) was purchased from Must Biotechnology Co.,Ltd. (Chengdu, China). Coumermycin A1 (HY-N7452) were obtained from MedChem Express (New Jersey, United States). The kits detecting malondialdehyde (MDA, RXJ302836R), glutathione (GSH, RXJ302694R), interleukin-IL6 (IL-6, RX302856R), interleukin-1β (IL-1β, RX302869R), and tumor necrosis factor-α (TNF-α, RX302058R) were obtained from RUIXIN BIOTECH (Quanzhou, China). Antibodies targeting aquaporin 4 (AQP4, 68448-1-Ig) was provided by Proteintech Group, Inc. (Wuhan, China). The antibody targeting hypoxia inducible factor-1 (HIF-1α, WL01607), JAK2 (WL02188), phospho-JAK2 (WL02997) were purchased from Wanleibio (Shenyang, China). The antibody targeting STAT3 (AF6294), phospho-STAT3 (AF3293) were purchased from Affinity Biosciences (Jiangsu, China). The antibody targeting β-actin (AC038), HRP-conjugated Goat anti-Mouse IgG (H + L) (AS003), HRP-conjugated Goat anti-Rabbit IgG (H + L) (AS014) were purchased from Affinity ABclonal (Wuhan, China). ROS probe Dihydroethidium (ID3560) was purchased from Beijing Solarbio Science and Technology Co., Ltd. (Beijing, China).

### 2.2 Animals

The animal study in this research was overseen by the Medical Ethics Committee at Chengdu University of Traditional Chinese Medicine (Approval number: 2023386), and conducted under the guidance of the national regulations on the management of laboratory animals and the manual for the management and use of laboratory animals. Fifty-four male Sprague-Dawley rats, aged 6 weeks and with a weight of 120 ± 20 g, were purchased from Chengdu Dashuo Experimental Animal Co., LTD. The animals were acclimated in a specific pathogen free (SPF) environment for 5 days, with a 12-hour dark/light cycle, humidity kept at 55% ± 5%, temperature at 25°C ± 1°C, and given standard diet and water freely available.

### 2.3 HACE model establishing

In order to replicate the conditions that lead to HACE, rats were exposed to a simulated high-altitude environment in an experimental hypobaric chamber (FLYDWC50-II C, Avic Guizhou Fenglei Aviation Armament Co., Ltd., China) equipped with pumps, chambers, oxygen supply pipes, connecting pipes, and communication systems (Jing et al., 2020; Wang et al., 2022). The experimental conditions were adjusted to replicate a 6,000 m altitude with an oxygen partial pressure of 9.6 kPa, while maintaining the ascent rate at 20 m/s. Once the target altitude was reached, the air intake and extraction rates, oxygen concentration, humidity, and temperature were kept constant at 15 L/min, 20 L/min, 20%, 55%, and 25°C, respectively. The duration of hypobaric hypoxia exposure was established at 48 h based on previous research (Wang et al., 2022). Rodents had unrestricted access to food and water during the hypobaric hypoxia exposure. The implementation of suitable measures led to a decrease in the distress felt by animals used in experiments.

### 2.4 Experiment design

Our experiment was divided into two phase: In the first phase, after a period of 5 days during which the rats were given adaptive feeding, thirty rats were separated into 5 groups randomly, with 6 rats in each group: (1) Sham group, (2) HACE model group, (3) EB 50 mg/kg + HACE (EB-L + HACE) group, (4) EB 100 mg/kg + HACE (EB-H + HACE) group, and (5) Dexamethasone 4 mg/kg + HACE (Dex + HACE) group. Dexamethasone, as a glucocorticoid, has anti-inflammatory properties and is effective for reducing the symptoms of HACE (Patir et al., 2012). Therefore, dexamethasone was used as a positive control drug based on its protective effect on HACE. The drug dosage and dissolution method for EB was chosen based on previous studies and well-established clinical dosing schedules (Wang et al., 2022). Rats in the Sham and HACE groups received normal intraperitoneal solvent injections, while the other 3 groups were given corresponding doses of EB or dexamethasone injections intraperitoneally for 3 days.

In the second phase of the research, coumermycin A1 (CA1), a JAK2 agonist (Gong L. et al., 2018), was employed to stimulate the JAK2 pathway in order to investigate the potential regulatory mechanisms of EB on JAK2/STAT3-mediated oxidative stress. The other 24 rats were randomly divided into 4 groups with 6 rats in each group as follows: (1) HACE model group, (2) EB 100 mg/kg + HACE (EB-H + HACE) group, (3) HACE + CA1 100 μg/kg (HACE + CA1) group, and (4) HACE + EB 100 mg/kg + CA1 100 μg/kg (EB-H + HACE + CA1) group. The choice of drug dose was based on previous studies (Gong L. et al., 2018; Wang et al., 2022). Rats in EB-H + HACE + CA1 group were intraperitoneally injected with CA1 1 h before EB injection. After the last injection, the rats in all groups except Sham group were placed in hypobaric anoxic chamber to replicate the HACE model.

**FIGURE 1:**
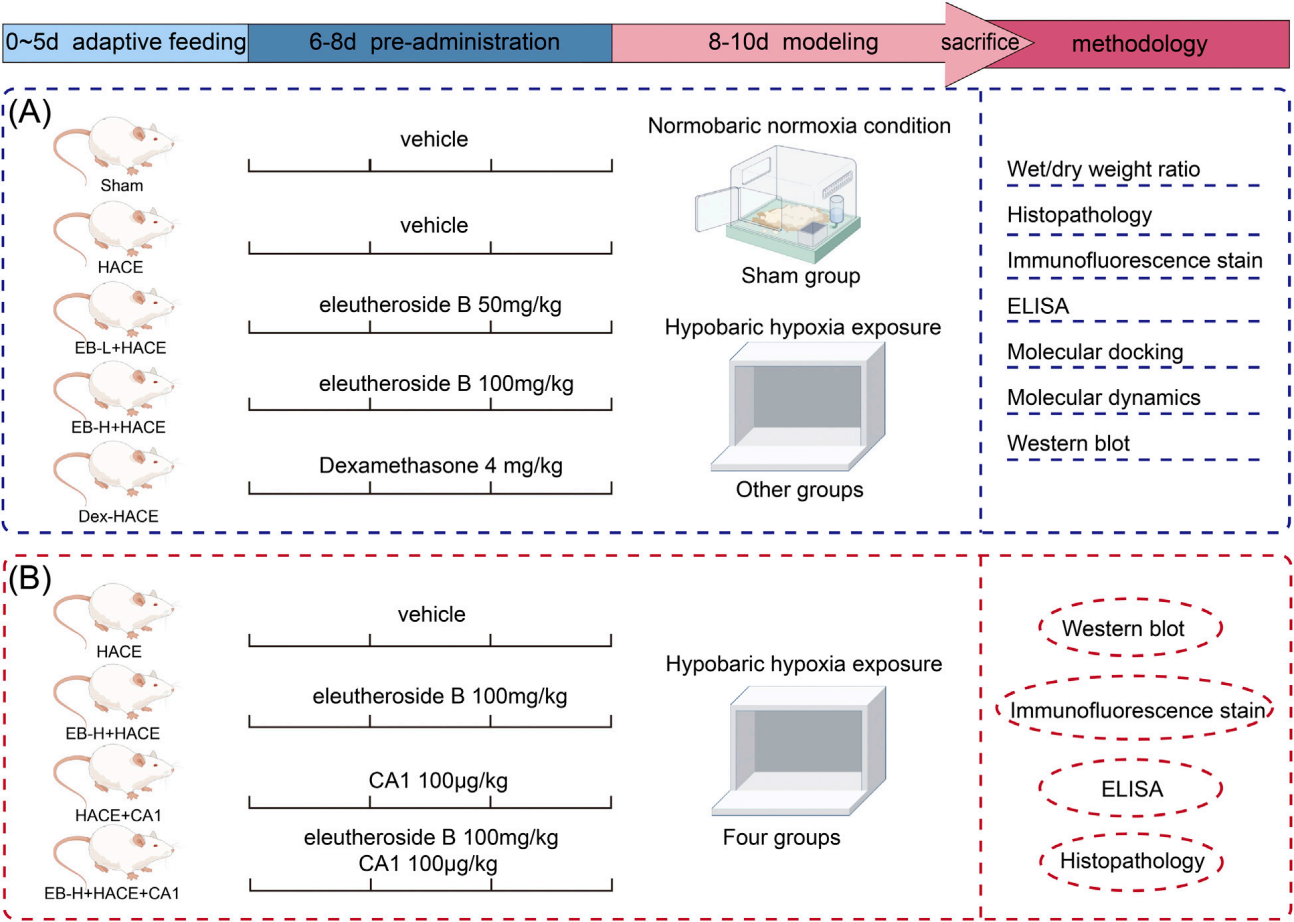
Schematic illustration of the experimental protocols of the study.

During the modeling, rats in Sham group were placed outside the normoxic chamber. After the modeling phase, the altitude conditions at 6,000 m were slowly returned to normal levels. After that, the rats were removed from the chamber and injected with sodium pentobarbital. During the treatment process, careful monitoring was conducted on the rats’ general wellbeing, such as their eating habits, digestion, excretion, fur quality, breathing and mental status. And the rats’ weights were documented on days 1, 4, 6, 8 and 10. The experimental procedure is shown in Figure 1.

### 2.5 Wet/dry weight ratio of brain tissue

After the sacrifice, the rat brain tissue was promptly dissected and any residual blood and moisture on the surface were delicately removed using absorbent paper. The processed left brain tissue was then placed on pre-weighed tinfoil and weighed collectively on an electronic analytical balance (YHK01SQP, sartorius, China). The recorded weight at this stage represented the wet weight (W) of the brain tissue. The sample was then dried in a constant temperature oven set at 60°C for 48 h until reaching a stable weight state. Following this, it was re-weighed the using the electronic analytical dry weight (D) of the brain tissue. In order to evaluate cerebral edema, the equation used to determine the moisture content of brain tissue is as follows: Brain tissue wet/dry ratio = (W-D)/W × 100%.

### 2.6 Histopathology of brain tissue

Hematoxylin-eosin staining and Nissellite staining were used to observe the structure, cell morphology and pathological changes of brain tissue under HACE. Brain tissue was fixed with 4% paraformaldehyde to maintain its morphological and structural integrity. The fixed brain tissue was treated with gradient dehydration and then embedded with paraffin wax to facilitate section. The 4-micron sections were acquired through the use of a microtome and then subjected to staining with hematoxylin-eosin and Nissellite solutions, followed by intermediate decolorization and differentiation processes. After staining, the structural features of the stained brain tissue and the morphology and distribution of the stained neurons were observed under a NanoZoomer S60 Digital Slicing Scanner (C13210-01, Hamamatsu Photonics Trading Co., Ltd., China).

### 2.7 Immunofluorescence staining of brain tissue

Reactive oxygen species (ROS) generated in the brain can be identified by utilizing the fluorescent dye DHE. The brain sections were deparaffinized, then subjected to boiling in a 0.01 M sodium citrate buffer (pH 6.0), followed by cooling at room temperature and three washes with PBS. Subsequently, the sections were exposed to 3% hydrogen peroxide to inhibit endogenous peroxidase activity, as well as 1% goat serum at room temperature. Following this, the samples were treated with 10 μM of ROS fluorescent probe DHE (Zhou et al., 2024) and left to incubate at 4°C overnight. The following day, they were washed with PBS and then exposed to enzyme-labeled secondary antibody at room temperature for 50 min before being stained with 4/,6-diamino-2-phenylindole. Finally, the fluorescence intensity of stained slices were obtained by 10× Leica DM6B fluorescence microscopy (DM6B, Leica, Germany) and analyzed by ImageJ software (Fang et al., 2024).

### 2.8 Oxidative stress and inflammation index in brain tissue

The brain tissue of rats was homogenized, centrifuged at 5,000 × g for 10 min, and the supernatant was obtained for analysis. The levels of inflammatory factors IL-6, IL-1β and TNF-α and oxidative stress index MDA, GSH in brain homogenate were detected by ELISA kit.

### 2.9 Molecular docking

The protein crystal structures for docking were obtained from the BrookHaven Protein Data Bank (PDB) database, with STAT3 and JAK2 having the respective PDB identifiers 6njs and 8ex2. The small molecule 3D structures were sourced from PubChem, followed by energy minimization conducted under the MMFF94 force field. In this research, the AutoDock Vina 1.2.3 software was utilized for conducting molecular docking (Burley et al., 2017). Prior to docking, the receptor proteins underwent processing with PyMol 2.5.22 to eliminate water molecules, salt ions, and small molecules (Kim et al., 2016). The docking box was positioned using the center of mass of the ligand in the original crystal as its focal point, with dimensions of 25*25*25 cubic angstroms. The small molecules and receptor proteins were then converted to PDBQT format for docking using AutoDock Vina 1.2.3 through the ADFRsuite 1.0 software toolkit (Trott and Olson, 2010). During the docking process, the global search exhaustiveness was set to 32, with all other parameters remaining at their default settings. Using the highest scoring output docking conformation as a guide, the bound conformation was determined, and subsequently, visual analysis was performed using PyMol 2.5.22 to interpret the docking results in this study.

### 2.10 Molecular dynamics (MD) simulation

Molecular dynamics simulations at the all-atom level were performed with AMBER 18 software, utilizing initial structures derived from docking small molecule and protein complexes (Salomon-Ferrer et al., 2013). Before running the simulation, the antechamber module and Hartree-Fock (HF) SCF/6–31G^*^ method in gaussian 09 software were used to calculate the charges of small molecules (Frisch et al., 2009; Wang et al., 2005). The GAFF2 force field was used to parameterize small molecules, whereas the ff14SB force field was employed for protein parameterization (Maier et al., 2015; Wang et al., 2004). The system was supplemented with hydrogen atoms using the LEaP module, and then a truncated octahedral TIP3P solvent box was added at a distance of 10 Å, along with Na^+^/Cl^−^ ions to maintain charge neutrality (Mark and Nilsson, 2001). Ultimately, the simulation produced topology and parameter files as its output.

The AMBER 18 program was utilized for conducting molecular dynamics simulations (Salomon-Ferrer et al., 2013). Prior to the simulation, the system underwent energy optimization with 2,500 steps of steepest descent method and 2,500 steps of conjugate gradient method. The temperature of the system was gradually increased from 0 K to 298.15 K over a period of 200 ps, while maintaining a constant heating rate and fixed volume. Subsequently, a simulation using an NVT ensemble was carried out for 500 ps at a constant temperature of 298.15 K to ensure uniform dispersion of solvent molecules throughout the solvent box. Equilibrium of the entire system was achieved through a 500 ps NPT simulation, followed by another NPT simulation under periodic boundary conditions for 100 ns with a truncated distance set at 10 Å. Long-range electrostatic interactions were computed using the PME method, while hydrogen bond lengths were constrained using the SHAKE method (Sagui and Darden, 1999). Temperature control was maintained using the Langevin algorithm with a collision frequency gamma of 2 ps^-1^ (Kräutler et al., 2015; Larini et al., 2007). The system pressure was held steady at 1 atmosphere, integration steps were configured to be 2 femtoseconds, and trajectories were recorded at intervals of every 10 ps for later analysis.

### 2.11 MMGBSA binding free energy calculations

The binding free energies between the protein and ligand were calculated using MM/GBSA for all systems (Genheden and Ryde, 2015; Hou et al., 2011; Rastelli et al., 2010). A MD trajectory of 45–50 ns was employed in this study to perform the calculations, utilizing a specific formula as follows:

$${\Delta G}_{bind}={\Delta G}_{\text{complex}}\;–\;\left({\Delta G}_{\text{receptor}}+{\Delta G}_{\text{ligand}}\right)$$

$$={\Delta E}_{\text{internal}}+{\Delta E}_{\text{VDW}}+{\Delta E}_{\text{elec}}{+\Delta G}_{\text{GB}}+{\Delta G}_{\text{SA}}$$

In the given equation, ${\Delta E}_{\text{internal}}$ represents the internal energy, ${\Delta E}_{\text{VDW}}$ denotes the van der Waals interaction, and ${\Delta E}_{\text{elec}}$ denotes the electrostatic interaction. The internal energies include Ebond energy, Eangle energy, and Etorsion energy, collectively known as solvation free energy. Here, G_GB_ represents polar solvation free energy and G_SA_ represents non-polar solvation free energy. In this study, we utilized the GB model for calculation (igb = 2) (Nguyen et al., 2013). The non-polar solvation free energy (Δ*G*_*SA*_) was calculated by multiplying the surface tension (γ) and solvent accessibility surface area (SA), with Δ*G*_*SA*_ = 0.0072 × ΔSASA (Weiser et al., 1999). Refraining from considering entropy change in this study is attributed to its high consumption of computational resources and limited accuracy (Hou et al., 2011).

### 2.12 Western blot

The brain tissue of the rats was extracted and treated with a solution called RIPA cracking retarded solution, which contained 1 mM phenyl methyl sulfonyl fluoride (PMSF), as well as protease inhibitor and phosphatase inhibitor. The mixture was homogenized at 4°C, then centrifuged at a force of 120,00 × g for 10 min to obtain the supernatant for measuring the total protein concentration. Separation gels of sodium dodecyl sulfate (SDS) were prepared in varying concentrations based on the molecular weights of the proteins. Following electrophoresis, it was subsequently moved onto a PVDF membrane. After the membrane was blocked with 5% skimmed milk-TRIS buffer for 60 min, the primary antibodies were left to incubate overnight at 4°C: anti-AQP4 (1:1,000), anti-HIF 1α (1:500), anti-JAK2 (1:1,000), anti-phospho-JAK2 (1:1,000), anti-STAT3 (1:500), anti-phospho-STAT3 (1:500), and anti-β-actin (1:10,000). And the PVDF membrane was washed and then exposed to the secondary antibody at room temperature for a period of 2 h on the following day. Upon completion of the incubation period, the proteins were detected using enhanced chemiluminescence technology and analyzed with the Bio-Rad image analysis system. Referred to as the internal control, β-actin was utilized for calculating the relative protein level using ImageJ-win 64 (National Institutes of Health, United States).

### 2.13 Statistical analysis

The data was analyzed and visualized using GraphPad Prism 8 (Graphpad, San Diego, CA) for statistical purposes. One-way ANOVA or a repeated two-way ANOVA followed by Tukey’s t-tests multiple comparisons were used to compare the groups. The data were presented as the mean ± Standard Deviation (SD), and statistical significance was defined as *p*-values below 0.05.

## 3 Results

### 3.1 Effect of EB pre-treatment on the HACE model

**FIGURE 2:**
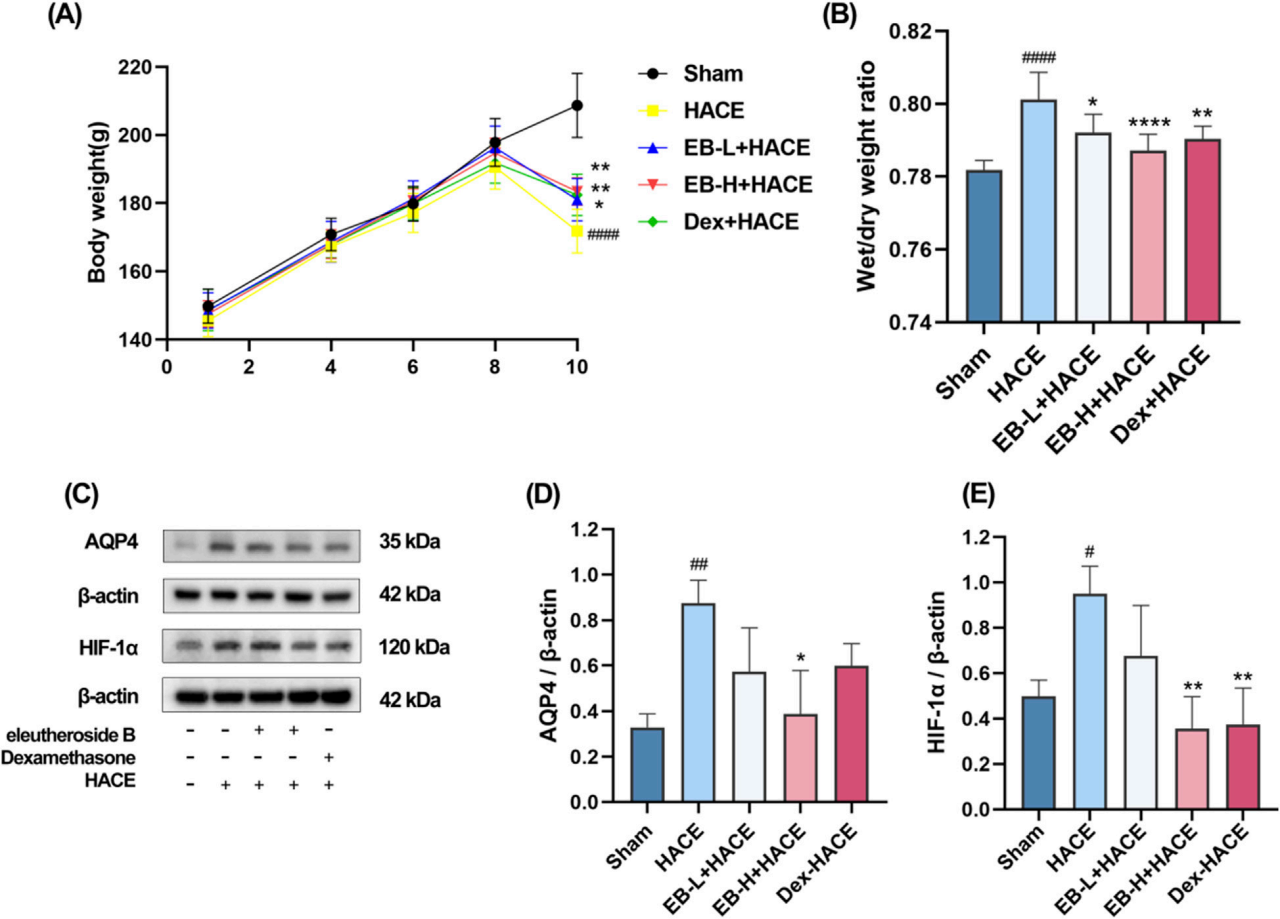
Effect of EB pre-treatment on the HACE model. **(A)** Weight changes in each group; **(B)** Brain water content in each group; **(C)** Representative Western blot images of AQP4 and HIF-1α; **(D–E)** The levels of AQP4 and HIF-1α protein expression were determined and normalized. Figure 2A was assessed using a repeated two-way ANOVA model with the Tukey’s *post hoc* multiple comparison test (n = 6). Figure 2B was assessed using one-way ANOVA with the Tukey’s *post hoc* multiple comparison test (n = 6). Figures 2D,E were assessed using one-way ANOVA with the Tukey’s *post hoc* multiple comparison test (n = 3). Data are mean ± SD; ^#^ *p* < 0.05 vs*.* Sham group; ^##^ *p* < 0.01 vs*.* Sham group; ^####^ *p* < 0.0001 vs*.* Sham group; ^*^ *p* < 0.05 vs*.* HACE group; ^**^ *p* < 0.01 vs*.* HACE group; ^****^ *p* < 0.0001 vs*.* HACE group.

In the experiment, the health status of rats in each group was observed. Before modeling, the rats in each group were in normal health. In the simulated environment with reduced pressure and oxygen levels, the rats had less food, water, defecation and urination, shortness of breath, and no significant changes in hair. The experiment involved recording the body weight of each group. As depicted in Figure 2A, there was no notable fluctuation in body weight within each group from day 1 to day 8. By day 10, the rats in the HACE group showed a noticeable decrease in body weight compared to those in the Sham group. This effect was mitigated based on dosage after pre-treatment with EB and Dex. These findings indicated that hypobaric hypoxia had adverse effects on the health and body weight of rats, which were alleviated by EB pretreatment.

Upon calculating and analyzing the dry-wet ratio, as illustrated in Figure 2B, it was observed that the model group exhibited a significantly higher brain water content compared to the Sham group. Moreover, both EB treatment and positive drug treatment effectively attenuated the HACE model-induced increase in brain water content, with this reduction demonstrating clear dose-dependent characteristics. HIF-1α serves as a pivotal transcription factor that modulates acute hypoxic gene expression changes and influences the organism’s physiological adaptation to hypoxic environments. AQP4 is integral to the development of brain edema and is a component of the ion channel complex responsible for water transport. Prior research has indicated that AQP4 and HIF-1α may have a synergistic effect in the pathogenesis of cerebral edema. Western blot analysis demonstrated an upregulation of HIF-1α and AQP4 expression in the HACE group, which was attenuated by EB pretreatment exclusively in the EB-H + HACE group (Figures 2C–E). These results indicated that EB pretreatment may significantly mitigate hypoxia and cerebral edema in HACE rats.

### 3.2 EB protected the brain tissue morphology and neuronal structure in the HACE model

**FIGURE 3:**
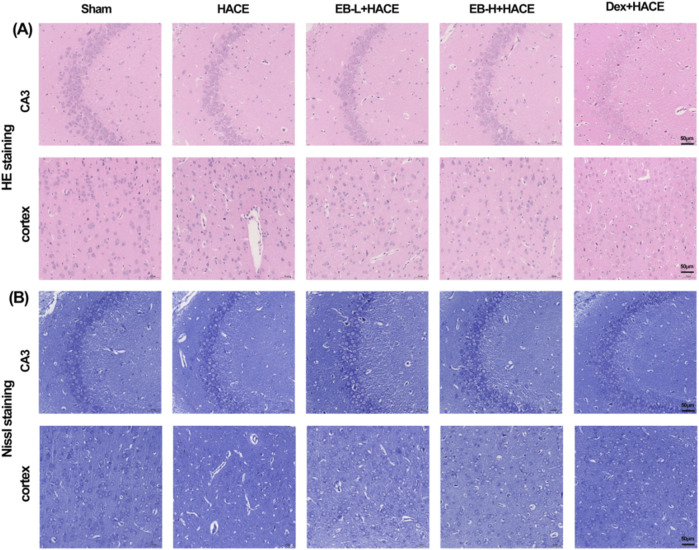
EB protected the brain tissue morphology and neuronal structure in the HACE model. **(A)** Representative photomicrographs were obtained using HE staining to show that EB prevented HACE-induced damage to neurons in the CA3 and Cortex areas. **(B)** Representative photomicrographs were obtained using Nissl staining to show that EB prevented HACE-induced damage to neurons in the CA3 and Cortex areas. Scale bar = 50 μm.

To evaluate the impact of EB on neuronal morphology and cell viability in a HACE model, we performed pathological analysis of hippocampal and cortical tissues using HE staining and Nissl staining techniques. The results in Figure 3A showed that under hypoxic conditions, the HACE group exhibited disorganized cell arrangement, cellular swelling, widened pericellular and perivascular space, intensified nuclear staining, and dilated blood vessels in the hippocampal CA3 region and cortical region. These pathological changes were significantly reduced after EB and Dex pre-treatment. Nissl staining results (Figure 3B) revealed that neurons in the control group displayed orderly arrangement and were filled with blue granular vesicles; moreover, the nucleolus and nuclear membrane were distinctly visible. In contrast, neurons from rats in the HACE group presented abnormal morphology, reduced viability, as well as blurred or dissolved nuclear membranes and nucleoli. In turn, EB or Dex pre-treatment notably alleviated these harmful effects. Clearly, hypobaric hypoxia could result in severe brain edema and tissue damage, while EB pre-treatment exerts a positive influence on ameliorating brain edema and preserving brain tissue morphology and neuronal structure.

### 3.3 EB inhibited the neuroinflammation in the HACE model

**FIGURE 4:**
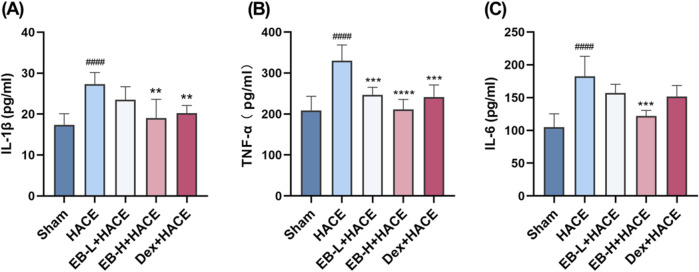
EB inhibited the neuroinflammation in the HACE model. **(A-C)** The concentration of IL-1β, TNF-α, and IL-6 levels in the brain tissue of each group. Statistical significance were assessed using one-way ANOVA with the Tukey’s *post hoc* multiple comparison test (n = 6). Data were presented as mean ± SD, ^**^ *p* < 0.01 vs. HACE group; ^***^ *p* < 0.001 vs. HACE group; ^****^ *p* < 0.0001 vs. HACE group; ^####^ *p* < 0.0001 vs. Sham group.

It has been generally recognized that altitude hypoxia can induce inflammation from previous studies of hypoxia signaling pathways. Previous clinical studies have shown that the increased levels of circulating pro-inflammatory cytokines lead to lung or brain edema. As HACE progresses, there is an increase in the expression of pro-inflammatory cytokines IL-1β, TNF-α, and IL-6 in the brain tissue (de Vries et al., 1996). As shown in Figure 4, Our study examined the influence of EB pro-inflammatory factor levels and observed that prior exposure to EB led to a reduction in the release of pro-inflammatory factors induced by hypoxia at low pressure plateau, with the inhibition being dependent on the dosage of EB. After pretreatment with EB, IL-1β, IL-6 and TNF-α overexpression was inhibited in the EB-H + HACE group. The results indicated that EB pre-treatment could effectively reduce the neuroinflammation in the HACE model.

### 3.4 EB reduced oxidative stress in the HACE model

**FIGURE 5:**
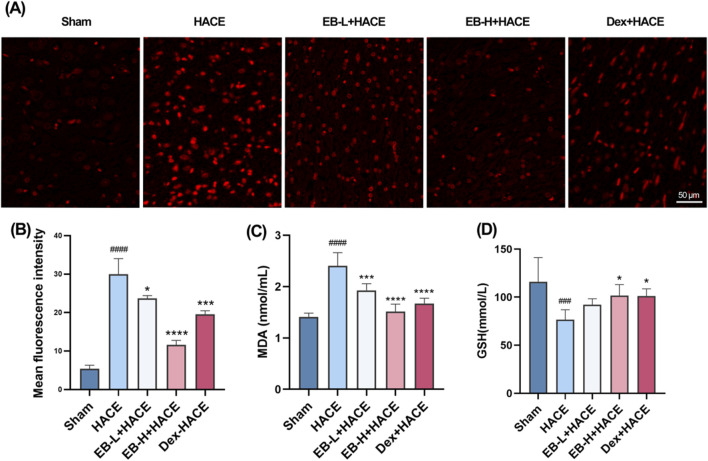
EB reduced oxidative stress in the HACE model **(A)** Representative images showing ROS-positive (red) in the cortex. Scale bar = 50 μm **(B)** Quantitation of ROS fluorescence intensity. **(C–D)** The concentration of MDA and GSH in brain tissue. Figure 5B was assessed using one-way ANOVA with the Tukey’s *post hoc* multiple comparison test (n = 3). Figures 5C,D were assessed using one-way ANOVA with the Tukey’s *post hoc* multiple comparison test (n = 6). Data are mean ± SD, ^###^ *p* < 0.001 vs*.* Sham group; ^####^ *p* < 0.0001 vs*.* Sham group; ^*^ *p* < 0.05 vs*.* HACE group; ^***^ *p* < 0.001 vs*.* HACE group; ^****^ *p* < 0.0001 vs*.* HACE group.

Previous studies have shown that oxidative stress can damage blood brain barrier and lead to extracellular edema. This suggests that oxidative stress induced by acute hypobaric hypoxia is an important factor leading to HACE. To demonstrate the protective effect of EB on rats with HACE, immunofluorescence technique and ELISA were used to investigate whether EB has the ability to prevent oxidative stress induced by hypobaric hypoxia. By immunofluorescence technique, we observed that the level of ROS in the cortical regions of HACE rats was significantly increased under hypobaric hypoxia, which is a typical manifestation of oxidative stress. The intervention of EB effectively inhibited the accumulation of ROS in Figures 5A,B. Furthermore, the concentrations of MDA and GSH in homogenized brain tissue were assessed using biochemical kits to further evaluate the anti-oxidatve stress activity of EB. As depicted in Figures 5C,D, the MDA levels in the brain tissue of HACE rats were found to have significantly increased, while the GSH levels exhibited a decrease compared to the control group. In both the EB-H + HACE and Dex + HACE group, there was a dose-dependent decrease in MDA and increase in GSH levels, with particular statistical significance observed in EB-H group. The findings indicated that EB has the potential to impede the pathological progression of HACE by mitigating oxidative stress.

### 3.5 Potential regulation of EB towards the JAK2/STAT3 signaling pathway

**FIGURE 6:**
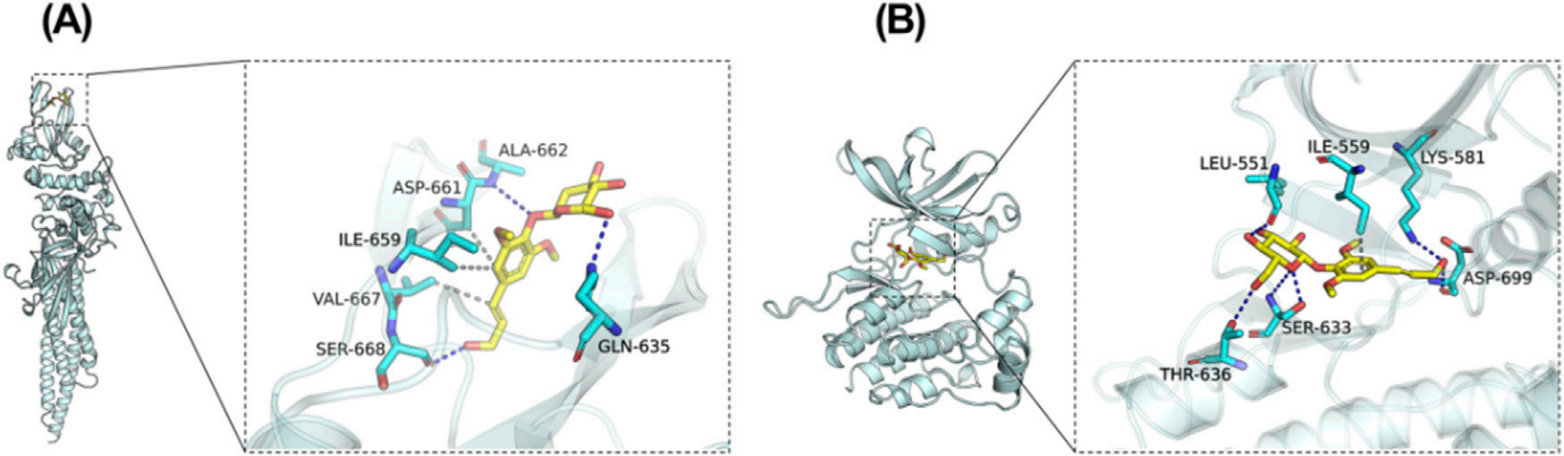
Potential regulation of EB towards the JAK2/STAT3 signaling pathway. Molecular Docking Analysis. **(A)** The binding interactions between JAK2 and EB **(B)** The binding interactions between STAT3 and EB. As elucidated through molecular docking studies, the left presents an overall view of the binding mode, the right provides a detailed partial view.

Revealing the potential mechanism of EB on HACE, we conducted a study on the interaction between EB and the kinase domains of JAK2 and STAT3 using molecular docking and molecular dynamics simulation. The 3D docking results of the two target proteins, JAK2 and STAT3, with EB are depicted in Figures 6A,B, respectively. During the docking process with JAK2 protein, EB formed stable hydrogen bonds with amino acid residues GLN-635, ALA-662, SER-668, and exhibited significant hydrophobic interactions with ASP-661, ILE-659, VAL-667. Similarly, when docking with STAT3 protein, EB established hydrogen bonds with multiple amino acid residues such as SER-633, ASP-699, THR-636, LEU-551, LYS-581 and engaged in hydrophobic interaction with ILE-559 residue.

As shown in Table 1, the affinity scores for compound EB binding to JAK2 and STAT3 proteins were −5.051 kcal/mol and −7.349 kcal/mol, respectively, revealing a possible interaction between EB and the protein kinase domain of both JAK2 and STAT3.

**TABLE 1:** The binding affinity scores of complexes (kcal/mol).

Target_name	Ligand_name	Docking_score
6njs-STAT3	Eleutheroside B	**−5.051**
8ex2-JAK2	Eleutheroside B	**−7.349**

**FIGURE 7:**
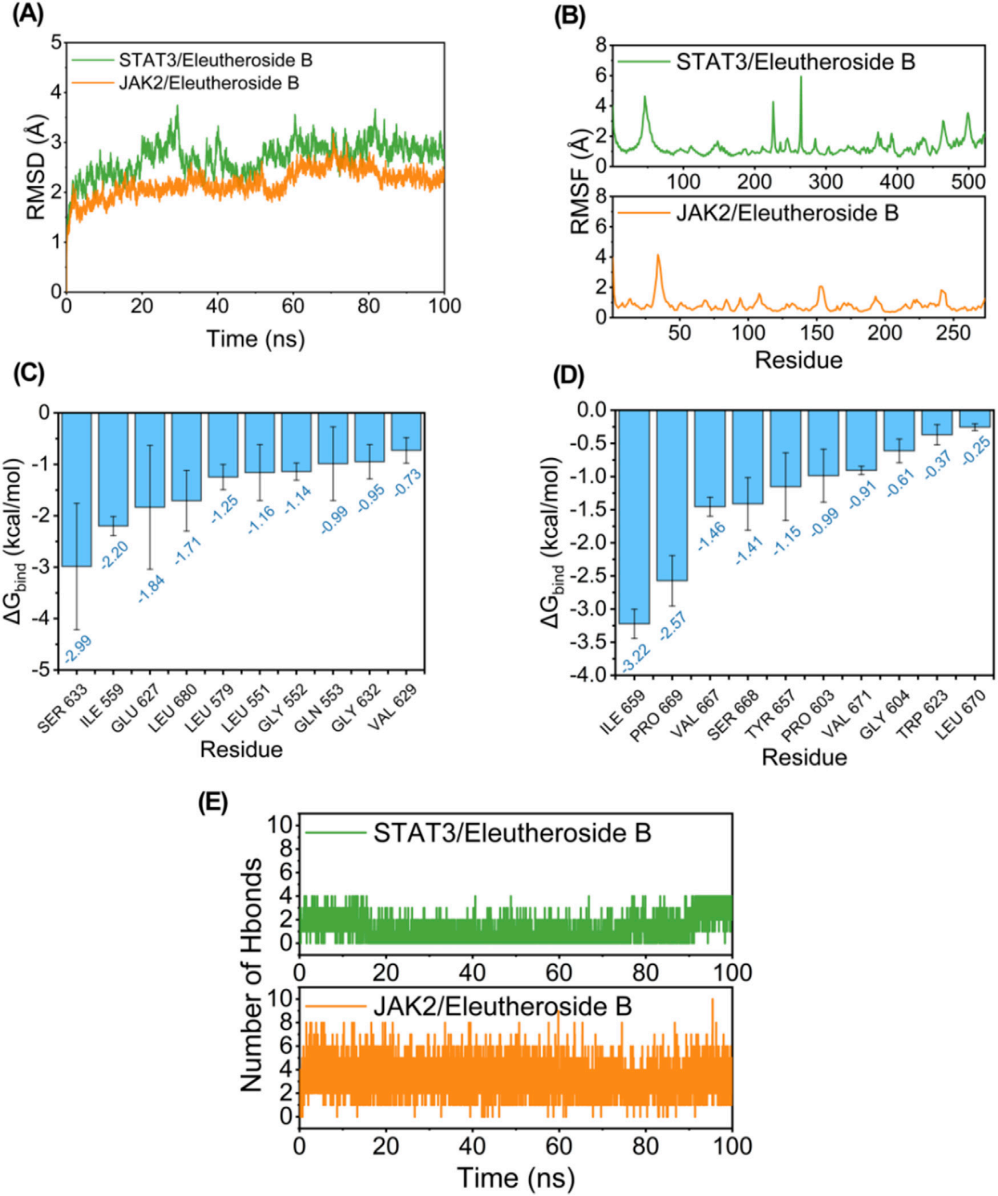
Potential regulation of EB towards the JAK2/STAT3 signaling pathway. Molecule dynamics **(A)** The Root Mean Square Deviation (RMSD) of the complexes over time during the molecular dynamics simulation. **(B)** The Root Mean Square Fluctuations (RMSF) calculated based on the molecular dynamics simulation trajectory. **(C–D)** The top 10 amino acids contributing to the binding of small molecules and proteins. **(E)** The variation in the number of hydrogen bonds between small molecules and proteins throughout the molecular dynamics simulation.

While conducting the simulation of molecular dynamics, as depicted in Figure 7A, both JAK2/EB and STAT3/EB complexes exhibited minimal RMSD fluctuations, indicating the stability of the complex structure and the appropriate setting of simulation parameters, thereby ensuring the reliability of the simulation results. Conversely, the JAK2/EB complex displayed higher stability in RMSD values, suggesting a superior binding effect. Root mean square fluctuation (RMSF) reveals the flexibility of proteins in simulations of molecular dynamics. Normally, drug binding to a protein reduces its flexibility and stabilizing the protein structure. As illustrated in Figure 7B, aside from localized regions of the protein, the RMSF value for JAK2 remained within 2 Å, signifying an exceptionally rigid main protein structure probably attributed to small molecule EB binding effects.

The MM/GBSA approach was utilized to estimate the binding free energy between the ligand and protein based on simulation trajectories from molecular dynamics, aiming to more accurately assess the interaction between EB and JAK2 or STAT3. The data in Table 2 reveals that the binding free energy of JAK2/EB and STAT3/EB is −31.19 ± 2.14 kcal/mol and −25.38 ± 1.85 kcal/mol, respectively, indicating a significant binding potential between EB and both JAK2 and STAT3 proteins. Notably, the binding affinity of JAK2/EB is particularly pronounced. Furthermore, utilizing the MM GBSA energy decomposition technique allowed for identification of the top 10 amino acid residues in JAK2 and STAT3 proteins that exerted maximal influence on small molecule EB binding is list in Figures 7C,D, thus representing key amino acid residues involved in EB-protein interaction. During simulation, in Figure 7E, hydrogen bond formation by JAK2/EB and STAT3/EB ranged from 0 to 8; notably, most hydrogen bonds formed by STAT3/EB were concentrated at 1, suggesting a relatively minor contribution of hydrogen bonding to stable binding with STAT3/EB. Conversely, for JAK2/EB, most hydrogen bonds were concentrated at 3 to 4, indicating a substantial contribution of hydrogen bond interactions to stable binding with JAK2/EB. By combining molecular docking outcomes with molecular dynamics simulations, it was found that EB demonstrates increased attraction to the kinase domain of JAK2, indicating its ability to establish strong connections with JAK2 and potentially hinder the activation of the JAK2/STAT3 signaling pathway.

**TABLE 2:** Free energy binding and energy components forecasted using MM/GBSA (kcal/mol).

System name	JAK2/Eleutheroside B	STAT3/Eleutheroside B
Δ*E*_vdw_	−36.66 ± 3.60	−33.00 ± 1.70
Δ*E*_elec_	−46.37 ± 7.64	−30.15 ± 1.71
ΔG_GB_	58.34 ± 8.26	42.15 ± 0.96
ΔG_SA_	−6.50 ± 0.35	−4.37 ± 0.12
ΔG_bind_	−31.19 ± 2.14	−25.38 ± 1.85

ΔE_vdW_: van der Waals energy.

ΔE_elec_: electrostatic energy.

ΔG_GB_: electrostatic contribution to solvation.

ΔG_SA_: non-polar contribution to solvation.

ΔG_bind_: binding free energy.

### 3.6 EB inhibited the JAK2/STAT3 signaling pathway in the HACE model

**FIGURE 8:**
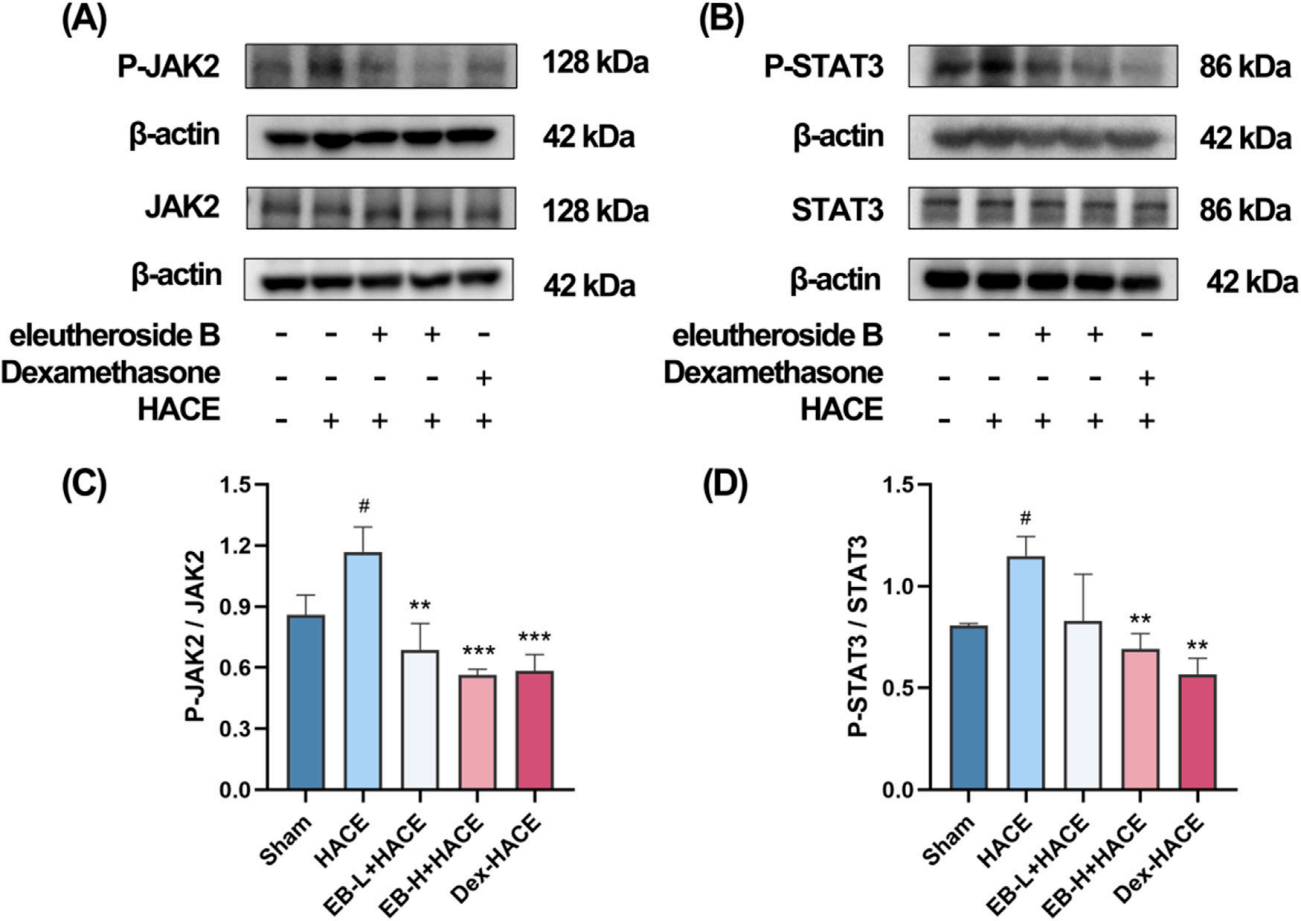
EB inhibited the JAK2/STAT3 signaling pathway in the HACE model. **(A)** Representative Western blot images of JAK2 and P-JAK2 in the brain tissue. **(B)** Representative Western blot images of STAT3 and P-STAT3 in the brain tissue. **(C–D)** The levels of P-JAK2/JAK2, P-STAT3/STAT3 protein expression, the calculation method for this data is defined as follows: the total protein expression level is determined by the ratio of phosphorylated protein expression to non-phosphorylated protein expression. Data were subjected to one-way ANOVA with Tukey’s *post hoc* multiple comparison test (n = 3). Data are mean ± SD; ^*^ *p* < 0.05 vs. HACE group; ^**^ *p* < 0.01 vs. HACE group; ^***^ *p* < 0.001 vs. HACE group; ^#^ *p* < 0.05 vs. Sham group.

Given the significant association between oxidative stress and the JAK2/STAT3 signaling pathway, we undertook additional research to investigate the effects of EB on this pathway in HACE rats. We used Western blot analysis to assess the protein expression related to the JAK2/STAT3 signaling pathway. As shown in Figure 8, although the overall protein expression levels of JAK2 and STAT3 did not show significant differences among the experimental groups, it is worth noting that the phosphorylated forms of JAK2 and STAT3 were found to be increased in the HACE group, indicating that the exposure to low oxygen levels triggered the JAK2/STAT3 signaling pathway in the brain tissue of rats suffering from HACE. Following pre-treatment with EB, the levels of protein phosphorylation in the JAK2/STAT3 signaling pathway exhibited a dose-dependent decrease. Especially, a significant and statistically meaningful decrease in phosphorylated expression of proteins associated with the JAK2/STAT3 signaling pathway was demonstrated for EB-H + HACE group. These findings suggest that EB has potential to alleviate HACE through inhibition of the JAK2/STAT3 signaling pathway.

### 3.7 The protective effect of EB partially depends on the JAK2/STAT3 signaling pathway in the HACE model

**FIGURE 9:**
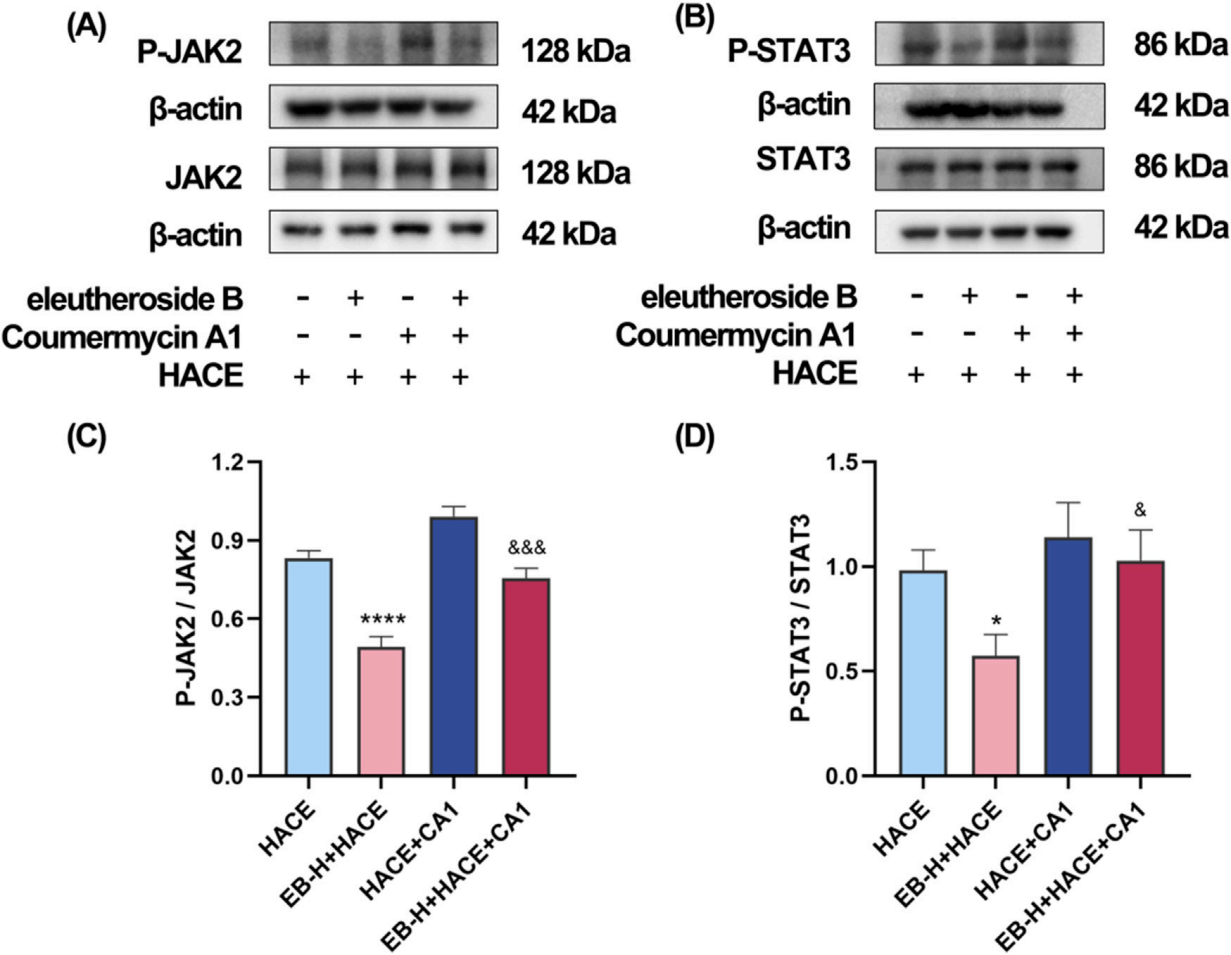
The protective effect of EB partially depends on the JAK2/STAT3 signaling pathway in the HACE model. **(A)** Representative Western blot images of JAK2 and P-JAK2 in the brain tissue. **(B)** Representative Western blot images of STAT3 and P-STAT3 in the brain tissue. **(C–D)** The levels of P-JAK2/JAK2, P-STAT3/STAT3 protein expression, the calculation method for this data is defined as follows: the total protein expression level is determined by the ratio of phosphorylated protein expression to non-phosphorylated protein expression. Data were subjected to a repeated two-way ANOVA model with the Tukey’s *post hoc* multiple comparison test (n = 3). Data are mean ± SD; ^*^ *p* < 0.05 vs. HACE group; ^***^ *p* < 0.001 vs. HACE group; ^&^ *p* < 0.05 vs. EB-H + HACE group; ^&&^ *p* < 0.01 vs. EB-H + HACE group; ^&&&^ *p* < 0.001 vs. EB-H + HACE group.

In order to investigate the dependency of the anti-oxidative effects of EB in HACE rat model on the inhibition of JAK2/STAT3 signaling pathway, a JAK2 specific agonist, CA1 was employed in this study. On the third day of EB pre-conditioning, CA1 was administered 1 hour prior to the experiment, followed by EB preconditioning and hypobaric hypoxia modeling. The Western blot analysis was used to assess the phosphorylation status of JAK2 and STAT3. The results in Figures 9A–D indicate that administration of EB resulted in a notable reduction in the phosphorylation of JAK2 and STAT3. However, compared with EB-H + HACE group, the phosphorylation levels in the brain tissue of the EB-H + HACE + CA1 group showed a significant increase. Taken together, these results indicate that the protective effect of EB partially depends on the JAK2/STAT3 signaling pathway.

### 3.8 EB`s inhibition of oxidative stress and neuroinflammation was dependent on the JAK2/STAT3 signaling pathway

**FIGURE 10:**
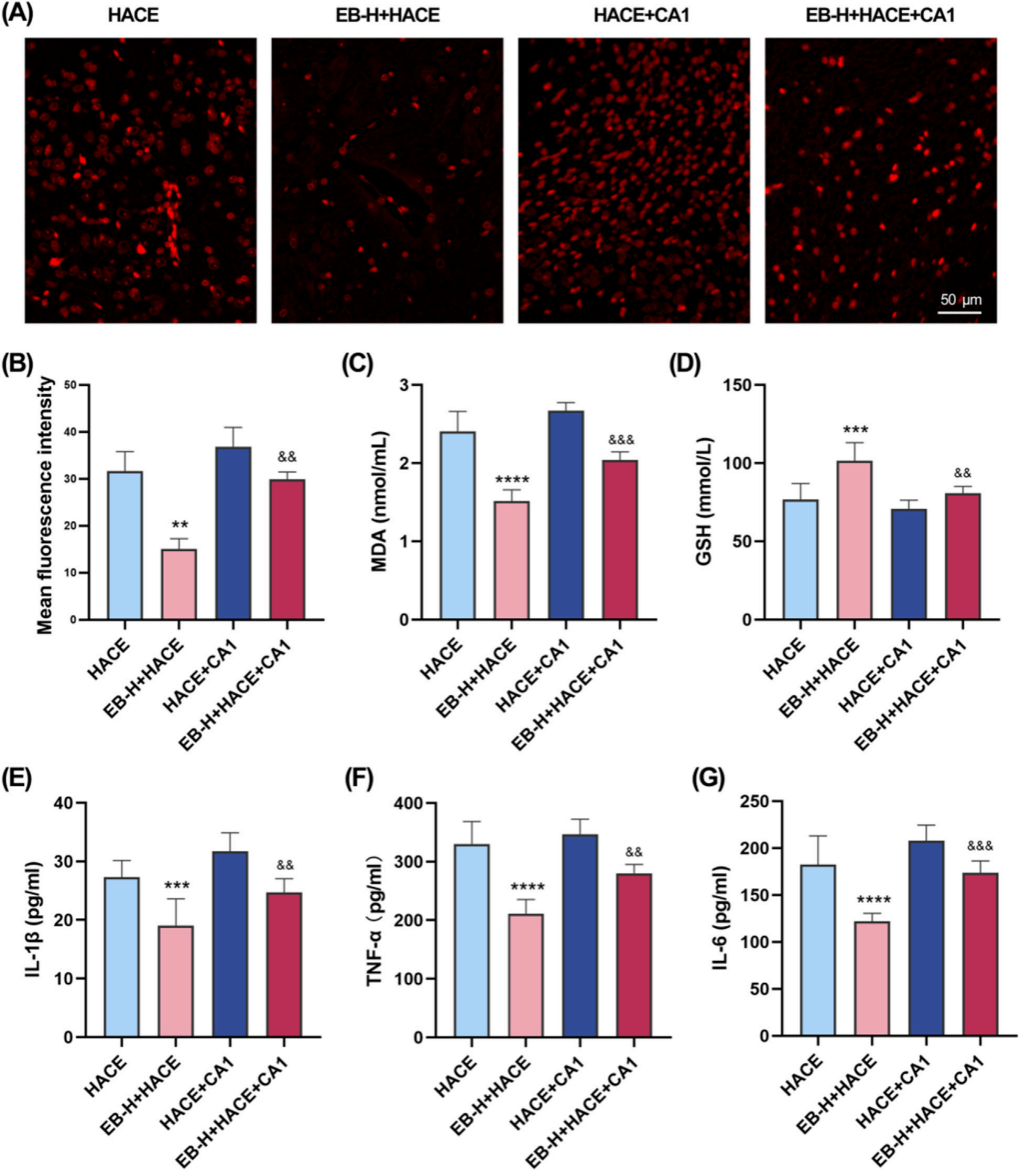
EB`s inhibition of oxidative stress and neuroinflammation was dependent on the JAK2/STAT3 signaling pathway. **(A)** Representative images showing ROS-positive (red) in the cortex. **(B)** Quantitation of ROS fluorescence intensity. **(C–D)** The concentration of MDA and GSH in brain tissue. **(E–F)** The concentration of IL-1β, TNF-α, and IL-6 levels in the brain tissue of each group. **(B)** was subjected to a repeated two-way ANOVA model with the Tukey’s *post hoc* multiple comparison test (n = 3). **(C–G)** were subjected to a repeated two-way ANOVA model with the Tukey’s *post hoc* multiple comparison test (n = 6). Data are mean ± SD; ^**^ *p* < 0.01 vs. HACE group; ^***^ *p* < 0.001 vs. HACE group; ^****^ *p* < 0.0001 vs. HACE group; ^&&^ *p* < 0.01 vs. EB-H + HACE group; ^&&&^ *p* < 0.001 vs. EB-H + HACE group.

Immediately following this, we further assessed whether the anti-oxidative stress and anti-inflammatory effects of EB affected the JAK2/STAT3 signaling pathway. As shown in Figures 10A,B, there was a marked increase in ROS fluorescence intensity in both the HACE group and the HACE + CA1 group. However, this effect was reversed by EB. In the HACE model, EB stimulation significantly decreased MDA activity in brain tissue, while GSH levels were notably increased. However, CA1 counteracted the EB-induced alterations in ROS, MDA and GSH. Similarly, EB substantially reduced the production of IL-1β, TNF-α, and IL-6 compared to the HACE group, whereas CA1 significantly elevated the expression of these inflammatory cytokines in brain tissue. These findings suggest that EB exerts its antioxidative stress and anti-neuroinflammatory effects through the JAK2/STAT3 signaling pathway.

### 3.9 EB’s protective effect on brain tissue and neurons in the HACE model relied on the JAK2/STAT3 signaling pathway

**FIGURE 11:**
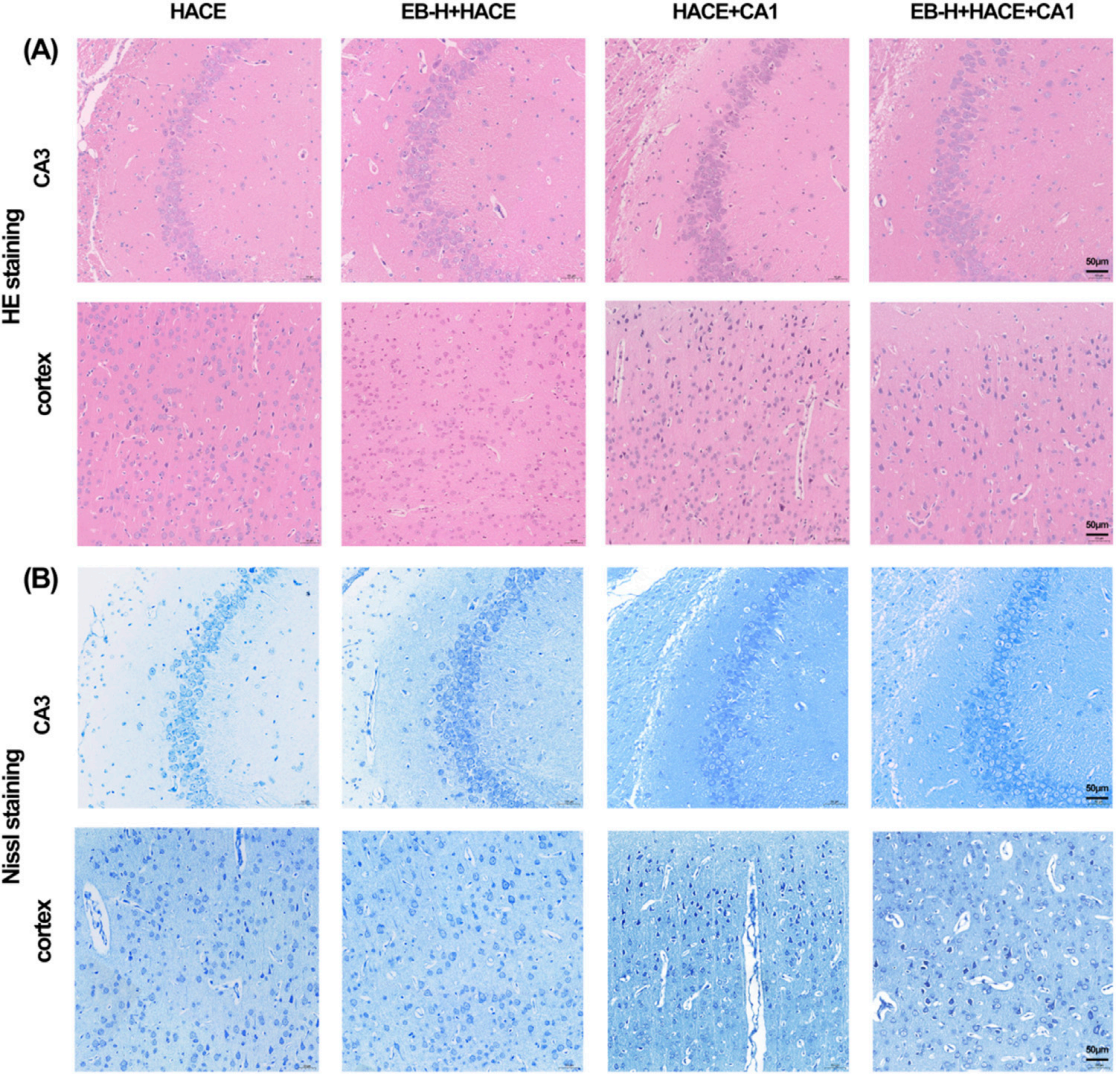
EB reversed the damage to brain tissue morphology and neuronal structure caused by the HACE + CA1 model. **(A)** Representative photomicrographs were obtained using HE staining to show that EB prevented HACE + CA1 induced damage to neurons in the CA3 and Cortex areas. **(B)** Representative photomicrographs were obtained using Nissl staining to show that EB prevented HACE + CA1 induced damage to neurons in the CA3 and Cortex areas. Scale bar = 50 μm.

In order to further validate the connection between EB’s therapeutic benefits and the JAK2/STAT3 signaling pathway, we performed a pathological examination of the hippocampus and cortex using HE and Nissl staining. The findings indicated that pre-treatment with EB notably ameliorated disordered cell arrangement, swelling, gap widening, nuclear staining enhancement, and vascular dilation in the hippocampal CA3 region and cortical region of the HACE group under hypoxia (Figure 11A). Furthermore, Nissl staining revealed that EB pre-treatment alleviated neuronal morphological abnormalities and decreased viability (Figure 11B). However, these positive effects were partially mitigated by CA1 intervention, suggesting that the therapeutic mechanism of EB on HACE may indeed be linked to its inhibition of JAK2/STAT3 signaling pathway.

## 4 Discussion

Acute cerebral edema at high altitudes presents with intense head pain, nausea, lack of coordination, and gradual impairment of awareness. Given the abrupt onset, elevated mortality rate, and complex pathophysiological mechanisms of HACE, it is imperative to develop a distinctive and effective therapeutic intervention swiftly. In recent years, the active ingredients in Chinese herbal medicine for the prevention and treatment of HACE have aroused the interest of many scholars. To the best of our knowledge, this study is the first to report that EB has been demonstrated to improve cerebral edema caused by high altitude hypobaric hypoxia, promoted the general health of rats, and successfully protected the integrity of brain architecture and neurons. Furthermore, EB was observed to mitigate oxidative stress and neuroinflammation associated with high-altitude hypoxia by inhibiting the JAK2/STAT3 signaling pathway. These results suggested that EB could be a new potential medication for preventing and treating HACE. Compared to the positive drug dexamethasone, EB, as a natural ingredient, exhibits fewer side effects and is considered safer for use, thus demonstrating significant potential for further development.

As elevation rises, the air becomes less dense and the amount of oxygen in the atmosphere decreases, which could result in disruptions to cell metabolism, abnormal circulation in the brain, heightened permeability of cerebral blood vessels, and ultimately lead to HACE. Consequently, individuals with HACE typically present with dilation, congestion, and edema of the cerebral base vessels in the brain surface area. As a result, a decrease in the partial pressure of oxygen triggers a sequence of cellular responses. The protein factor HIF-1, known as Hypoxia inducible factor-1, has the ability to interact with DNA and is essential in transmitting signals related to decreased oxygen levels (Semenza, 2001). The HIF-1 transcription factor consists of two subunits, α and β, with HIF-1α being the isoform that responds to hypoxic conditions. Under low oxygen levels, there is an increase in the expression and activity of HIF-1α, resulting in its binding to HIF-1β. This combination attaches to the enhancer or promoter region of specific genes, thereby stimulating gene expression. Additionally, hydroxylation of HIF-1α is suppressed under hypoxic conditions, resulting in elevated overall levels of HIF and increased transcriptional activity that stimulates the expression of genes related to hypoxia (Wu et al., 2022). As a result, HIF-1α acts as an essential marker for evaluating the presence of hypoxia (Stolze et al., 2002). Furthermore, there is growing evidence indicating the crucial role of aquaporin 4 (AQP4) in maintaining brain water balance. Aquaporins, also known as AQPs, are specialized proteins found on cell membranes that are necessary for regulating the amount of water inside cells. In the brain, AQP4 is the most prevalent type of aquaporin. Research has shown that AQP4 is significantly involved in the formation of cerebral edema (Papadopoulos and Verkman, 2007). The distribution and quantity of AQP4 molecules in the brain are controlled by complex mechanisms, including interactions with different proteins, changes in hypoxia and osmotic pressure, ammonia production, and transcriptional regulation (Gunnarson et al., 2004). AQP4 is closely linked to water movement within the brain, which impacts how fluid accumulates or dissipates within brain tissue (Bloch and Manley, 2007). Research has indicated that mice without AQP4 exhibit enhanced neurological recovery and decreased brain swelling after experiencing a focal ischemic stroke (Manley et al., 2000). Therefore, AQP4 serves as an essential indicator for assessing the occurrence of brain edema. Our study found that exposure to high-altitude conditions increased the levels of HIF-1α protein, tissue water content, and AQP4 protein expression in rat brain tissues, consisting with previous reports. These changes indicated the development of brain edema due to hypoxia, confirming the successful establishment of HACE in our rat model. Similar to its ability to decrease brain edema in other types of brain injury (Tan et al., 2021), pre-treating with EB effectively reduced the increase in brain water content caused by HACE. In addition, it resulted in a decrease in the levels of HIF-1α and AQP4 proteins, indicating that EB has effects on reducing brain edema effects in HACE rats.

Neuroinflammation has been implicated as a contributor to cerebral edema, a major factor in the neurologic morbidity associated with HACE (Sarada et al., 2015). It has been shown that decreased levels of oxygen can affect the production of mRNA and the release of proinflammatory cytokines such as IL-1β, IL-6, and TNF-α, resulting in the activation of signaling pathways for an inflammatory response to hypoxia (Choi et al., 2017; Wang et al., 2018). In cases of clinical HACE, it is thought that pro-inflammatory cytokines including IL-1β, IL-6, and TNF-α have significant involvement (de Vries et al., 1996). Consisting with clinical observations indicating heightened levels of these cytokines in individuals suffering from acute mountain sickness (Lundeberg et al., 2018; Wang et al., 2018), our study revealed a notable rise in the concentrations of IL-1β, IL-6, and TNF-α in the cerebral tissue of HACE model rats. Furthermore, reaching high altitudes can lead to acute hypobaric hypoxia, which can cause an overproduction of oxygen free radicals within the body. These radicals possess the ability to harm cell membranes, enzymes, and DNA, potentially resulting in cell dysfunction or death (Chakraborty and Roychoudhury, 2022). In addition, the hypoxic conditions appear to induce oxidative stress by elevating the generation of ROS within the mitochondrial electron transport chain, leading to a disruption in the oxidation equilibrium (Mohanraj et al., 1998). As a result, oxidative stress induced by hypoxia is considered a primary mechanism of acute HACE and has been supported by animal studies. Exposure of rats to hypobaric hypoxia has been found to result in elevated levels of oxidative stress markers ROS and MDA in brain tissues, accompanied by a reduction in the level of GSH, a critical cytoprotective agent against free radical damage. This indicates that HACE can induce an oxidative stress response in rats, leading to a significant decrease in antioxidant capacity (Gong G. et al., 2018; Himadri et al., 2010). Furthermore, neuroinflammation is intricately associated with the activity of innate immune cells within the CNS, particularly microglia and astrocytes. Upon activation, these neuroglial cells secrete a range of proinflammatory mediators and oxygen free radicals, which may play a role in maintaining tissue homeostasis or contribute to the pathogenesis of HACE. Our findings provide additional evidence supporting this assertion. The anti-inflammatory and antioxidant properties of EB may offer potential benefits for the prevention and treatment of HACE.

Current research is concentrating on the potential involvement of various inflammatory mediators and oxygen free radicals in HACE. The JAK2/STAT3 signaling pathway, a recognized target of inflammatory cytokines such as IL-6 (Boengler et al., 2008), plays a pivotal role in modulating oxidative stress and mitochondrial function, particularly concerning the overproduction of ROS (Yang et al., 2013). Consequently, our mechanistic study is centered on investigating the JAK2/STAT3 pathway. The JAK/STAT pathway has been recently discovered as a mechanism for signal transduction that specifically reacts to a wide variety of external regulatory signals. This pathway involves receptors related to tyrosine kinase and two protein families, with Janus kinase 2 (JAK2) and signal transducer and agonist of transcription 3 (STAT3) being the most widely affected forms in response to cellular environmental stimulation or stress adaptation (Rane and Reddy, 2000). Regarded as an important membrane-nuclear signaling pathway, the JAK2/STAT3 signaling pathway is capable of responding to various different signaling pathways. When activated and phosphorylated by cytokine receptors, JAK2 subsequently activates STAT3 in the cytoplasm, leading to its movement into the nucleus for controlling transcription of target downstream genes. Multiple research studies have confirmed the specific role of the JAK2/STAT3 in the CNS, and its involvement in CNS development, including promoting the growth, survival, and differentiation of neurons (Amantea et al., 2011; Bjørbaek and Kahn, 2004). Under normal conditions, the JAK2/STAT3 signaling pathway remains mostly inactive. However, it is only activated in response to ischemic or hypoxic stimulation and exhibits prolonged high levels of expression in neurons neighboring injured brain tissues as a result of different pathogenic factors including vascular remodeling, apoptosis, autophagy, inflammation, and oxidative stress (Li et al., 2003). Research has indicated that the activation of STAT3 may contribute to the generation and discharge of pro-inflammatory agents, as well as the initiation of oxidative stress damage from hydrogen peroxide, ultimately impeding brain recovery (Zhou and Yu, 2013). In our research, we noticed a rise in the levels of p-JAK2 and p-STAT3 in the brain tissue, suggesting that a plateau hypobaric hypoxia environment may trigger oxidative stress response by activating the JAK2/STAT3 pathway.

Consequently, our findings indicate that EB exerts its antioxidant stress and anti-neuroinflammatory effects through modulation of the JAK2/STAT3 signaling pathway. In future studies, we will use gene knockout mice and primary neuron cells in order to further investigate the molecular biological mechanisms underlying the interaction between EB and the JAK2/STAT3 signaling pathway. Furthermore, more comprehensive investigations are needed to assess the clinical potential of EB combining with pharmacokinetics, pharmaceuticals and clinical pharmacology.

## 5 Conclusion

This research has examined the significant impact of EB in reducing high-altitude cerebral edema, showing its positive effects in decreasing oxidative stress and neuroinflammation. These results indicate that EB may effectively protect rats from HACE by regulating the JAK2/STAT3 signaling pathway. This regulation mechanism effectively reduces oxidative stress and neuroinflammation caused by hypoxia at high altitudes, thus alleviating symptoms of HACE. The additional experimental results further support the dual anti-inflammatory and anti-oxidative effects of EB, a natural compound, highlighting its potential significance in addressing inflammatory and oxidative stress issues associated with high altitude hypoxia. These findings not only provide a scientific basis for using EB to treat high altitude cerebral edema but also offer new insights and opportunities for developing natural pharmaceuticals in the future.

## Data Availability

The raw data supporting the conclusions of this article will be made available by the authors, without undue reservation.
